# Visceral leishmaniasis risk clusters on Brazil’s borders: 2013 to 2022

**DOI:** 10.1590/0037-8682-0020-2025

**Published:** 2026-02-16

**Authors:** William da Costa Moreira, Gustavo Cezar Wagner Leandro, Helder Ferreira, Rosane Meire Munhak Silva, Demilto Yamaguchi da Pureza, Catchia Hermes-Uliana, Neide Martins Moreira

**Affiliations:** 1 Universidade Estadual do Oeste do Paraná, Programa de Pós-Graduação em Saúde Pública em Região de Fronteira, Foz do Iguaçu, PR, Brasil.; 2 Universidade Estadual de Maringá, Maringá, PR, Brasil.; 3 Universidade Federal do Amapá, Programa de Pós-Graduação em Ciências da Saúde, Macapá, AP, Brasil.; 4 Universidade Federal do Mato Grosso do Sul, Curso de Enfermagem, Três Lagoas, MS, Brasil.

**Keywords:** Visceral leishmaniasis, Border areas, Border health, Spatial analysis, Time series studies

## Abstract

**Background:**

Visceral leishmaniasis remains a significant public health burden in the Americas, particularly in Brazil and its border regions. This study aims to analyze the temporal trends and spatial clusters of visceral leishmaniasis in Brazilian border municipalities in 2013-2022.

**Methods:**

An ecological study was conducted, using secondary population-based data. Joinpoint regression was used to assess temporal trends and estimate the annual percentage change (APC). Kulldorff’s scan statistics identified spatial clusters and relative risks (RR).

**Results:**

During the period, 677 visceral leishmaniasis cases were reported in the border municipalities (average annual incidence rate: 0.58). The highest cumulative IR was observed among children and adolescents (10.05, 95% CI: 9.02-11.16), males (7.17, 95% CI: 6.50-7.88), and in the Central Arc 13.55, 95% CI: 12.25-14.95). Central Arc showed a decreasing trend from 2013-2020 (APC: -11.65%, 95% CI: -27.28-5.91) and an increasing from 2020-2022 (APC: 55.76%, 95% CI: 6.70-101.96), with RR rising 21.059 (2016-2019) to 34.310 (2020-2022). Persistent high-risk clusters were identified in the North Arc (RR: 100.529-156.040) and Central Arc (RR: 20.716-34.310), alongside a high-risk cluster in the South Arc near the Foz do Iguaçu triple border (RR: 3.466).

**Conclusion:**

This study revealed that visceral leishmaniasis in the Brazilian border region presents heterogeneous spatial and temporal patterns. Achieving effective disease control by 2030 will require strengthened health interventions and coordinated multilateral collaboration among neighboring countries, focusing on high-risk areas.

## INTRODUCTION

Visceral leishmaniasis (VL), a vector-borne zoonosis, is a significant public health problem in several countries. Globally, the annual incidence of new cases ranges from 50,000 to 90,000, with the majority concentrated in Brazil, particularly in the Northeast region^1^ ^-^ ^3^. Autochthonous transmission to humans has been reported in 21 of Brazil’s 26 states and in the Federal District, and its widespread geographic distribution remains a major challenge for surveillance and control efforts at both national and regional levels².

The spatial spread of VL has been accompanied by significant shifts in transmission patterns, with cases occurring in major urban centers and peri-urban areas^4^. Contextual factors such as poor nutrition, environmental and land-use changes, human migration, insufficient sanitation services and inadequate control over vectors and reservoirs possibly contribute to these evolving patterns^4^.

Building on this complex scenario, the Pan American Health Organization (PAHO) approved a regional plan of action aiming to reduce morbidity and mortality from leishmaniasis in the Americas by 2030^5^. Notable progress has been made in early case detection; however, persistent challenges remain, particularly in remote and hard-to-reach areas where health care infrastructure remains inadequate^6^ ^,^ ^7^.

Moreover, Brazil's international borders are strategic areas for epidemiological studies of VL^8^ ^-^ ^10^ due to the intense movement of people and animals between countries, which facilitates the spread of the disease^7^ ^-^ ^11^. These regions share socioeconomic and environmental vulnerabilities, underscoring the importance of spatial and temporal analysis to support more effective and integrated public policies^11^ ^-^ ^14^.

VL has been reported in countries bordering Brazil, including Paraguay, which surpassed 4,000 cases in 2022^15^. Between 2007 and 2018, the disease spread widely in Colombia, particularly in socially vulnerable areas^16^. The spatial and temporal dynamics of VL in Brazil have been thoroughly investigated to understand the disease’s expansion within the country over recent decades^7^ ^,^ ^11^ ^,^ ^14^. Previous studies have identified high-risk areas for VL along Brazil’s border regions; however, these findings were based on national-level analyses, which may overlook smaller, localized risk areas within border municipalities.

This study aims to analyze temporal trend variations and identify high-risk spatial clusters of VL in Brazilian border municipalities between 2013 and 2022.

## METHODS

### Study design and period

This ecological temporal and spatial study utilized population-based epidemiological surveillance data from 586 border municipalities as the unit of analysis. Newly diagnosed and reported cases of VL between 2013 and 2022 were aggregated according to municipality of residence. The study was reported in accordance with the Strengthening the Reporting of Observational Studies in Epidemiology (STROBE) guidelines.

### Study location

Brazil covers an area of 8,510,417 km² and has a population of 203,080,756, resulting in a density of 23.86 individuals per km² ^17^. Among Brazil’s 27 states, 11 are contiguous with Argentina, Uruguay, Paraguay, Bolivia, Peru, Colombia, Venezuela, Guyana, and Suriname^18^. The Brazilian border area is divided into three arcs, comprising the following states: the North Arc (Acre, Amapá, Amazonas, Pará, and Roraima), the Central Arc (Mato Grosso, Mato Grosso do Sul, and Rondônia), and the South Arc (Paraná, Rio Grande do Sul, and Santa Catarina). The Border Arcs encompass 586 municipalities located up to 150 km inland from Brazil’s land frontiers, distributed as follows: 416 in the South Arc, 101 in the Central Arc, and 69 in the North Arc^17^ ^,^ ^19^.

### Data source

In June 2024, case-level reports of VL were extracted from Brazil’s national notifiable diseases system (*Sistema de Informação de Agravos de Notificação*, Sinan), a database maintained by the Department of Informatics of the Unified Health System (DATASUS) and provided by the Brazilian Ministry of Health^19^. These reports contain patient demographics, clinical symptoms and signs, municipality of residence, laboratory results, and clinical outcomes^19^. 

Municipal population estimates were based on data provided by the Brazilian Ministry of Health^20^. The digital cartographic grid (shapefile format) of municipal boundaries, based on the Universal Transverse Mercator (UTM) projection and the Geodetic Reference System - SIRGAS 2000, was obtained from the Brazilian Institute of Geography and Statistics (*Instituto Brasileiro de Geografia e Estatístic*a, IBGE)^21^.

### Study variables

Confirmed cases of VL reported between 2013 and 2022 were included based on the municipality of residence and stratified by sex (female and male) and age group: children and adolescents (≤19 years), adults (20 to 59 years), and older adults (≥60 years), across the 586 municipalities located in the border area. The incidence rate of VL was calculated using population estimates provided by the Ministry of Health^20^. Cumulative and annual incidence rates (per 100,000 inhabitants) were calculated for municipalities (n = 586) and Border Arcs (North, Central, and South). Cumulative incidence rates were estimated using the total number of cases during the period and the estimated resident population at the end of the observation period (2022) as the denominator. Annual incidence rates were calculated based on new cases and population estimates for each corresponding year (t).

$$Cumulative\;incidence\;rate=\;\left(\frac{Total\;number\;of\;cases\;during\;the\;period}{Estimated\;population\;at\;the\;final\;of\;the\;period\;(2022)}\right)\times 100,000$$

$$Annual\;incidence\;rate\;t=\;\left(\frac{Number\;of\;new\;cases\;in\;year\;t}{Estimated\;population\;in\;year\;t}\right)\times 100,000$$

### Statistical analysis

Joinpoint regression was used to analyse the temporal trend of VL incidence rates, with the year of notification as the independent variable. The annual number of cases was modeled as the dependent variable, incorporating the logarithm of the population as an offset. Analyses were stratified by Border Arc (North, Central and South). The analysis began with a model assuming no joinpoints and subsequently tested for the presence of a single joinpoint. A minimum of two years of data was required for each segment, meaning that no joinpoints could be placed within the first two years, the last two years, or between segments shorter than two years. To address the presence of non-constant variance, the Poisson variance option was applied, assuming that case count variability follows the properties of a Poisson distribution. The best-fitting model was selected using the Weighted Bayesian Information Criterion (WBIC). Trends for each segment were expressed as annual percent change (APC) and classified as increasing (p < 0.05 and 95% CI > 0), decreasing (p < 0.05 and 95% CI < 0), or stationary (p ≥ 0.05). All analyses were conducted using the Joinpoint Regression Program (version 5.2.0.0^22^, with the significance level set at α = 0.05.

Spatial relative risk of infection by VL was identified using the Kulldorff spatial scan technique, implemented through SaTScan (version 10.2) software. A matrix was constructed with VL case counts, inhabitants and the geographical coordinates (longitude and latitude) of each municipality. The purely spatial scan, based on a Poisson distribution, was applied scanning a circular window, requiring a minimum of two cases and covering up to 50% of the total population. The years were segmented into the periods 2013-2015, 2016-2019, and 2020-2022. This analysis compared the number of cases observed with the number expected in each spatial cluster, giving a relative risk (RR) of infection^23^. In this process, SaTScan uses a coordinate’s information to identify the closest locations to the center of each constructed circle based on Euclidean distances, sequentially finding the nearest neighbors until the maximum window size is reached. Choropleth maps were produced using the cartographic base of Brazil’s border-area municipalities provided by IBGE and processed in QGIS (version 2.18.2) software.

### Ethical considerations

As the study used only anonymized secondary data, ethical approval was not required, in accordance with Resolution No. 466/2012 of the Brazilian National Health Council.

## RESULTS

From 2013 to 2022, a total of 677 VL cases were reported in Brazil’s border area, corresponding to an average annual incidence rate of 0.58 cases per 100,000 inhabitants. Among these VL cases, 62.92% occurred in males, with a cumulative incidence rate of 7.17 cases per 100,000 inhabitants (95% CI: 5.30 to 7.88). Children and adolescents were the most affected, representing 51.70% of cases, with a cumulative incidence rate of 10.05 per 100,000 inhabitants (95% CI: 9.02 to 11.16). The Central Arc accounted for most VL cases (58.49%), followed by the North (34.12%) and South Arcs (7.39%). Cumulative incidence rate was highest in the Central Arc (13.55 cases per 100,000 inhabitants, 95% CI: 12.25 to 14.95), followed by the North (9.42 cases per 100,000 inhabitants, 95% CI: 8.24 to 10.72) and the South (0.76 cases per 100,000 inhabitants, 95% CI: 0.57 to 1.01) (Table 1).

**TABLE 1:** Confirmed cases and cumulative incidence rate (per 100,000 inhabitants) of visceral leishmaniasis and population in municipalities in Brazil’s border area, 2013-2022.

Variables	Confirmed cases^a^ n (%)	Population^b^ n	Cumulative incidence rate (per 100,000 inhabitants) (95%CI)^a^
**Sex**			
Female	251 (37.08)	5,971,714	4.20 (3.70 to 4.75)
Male	426 (62.92)	5,943,544	7.17 (6.50 to 7.88)
**Age group**			
Children and adolescents	350 (51.70)	3,483,978	10.05 (9.02 to 11.16)
Adults	250 (36.93)	6,669,960	3.75 (3.30 to 4.24)
Older adults	77 (11.37)	1,761,320	4.37 (3.45 to 5.46)
**Border Arc**			
North	231 (34.12)	2,451,916	9.42 (8.24 to 10.72)
Central	396 (58.49)	2,923,034	13.55 (12.25 to 14.95)
South	50 (7.39)	6,540,308	0.76 (0.57 to 1.01)
**Total**	**677**	**11,915,258**	**5.68 (5.26 to 6.13)**

**Source:** prepared by the authors from Datasus data. **Legend:** ^a^ From 2013 to 2022. ^b^ 2022.

**FIGURE 1:**
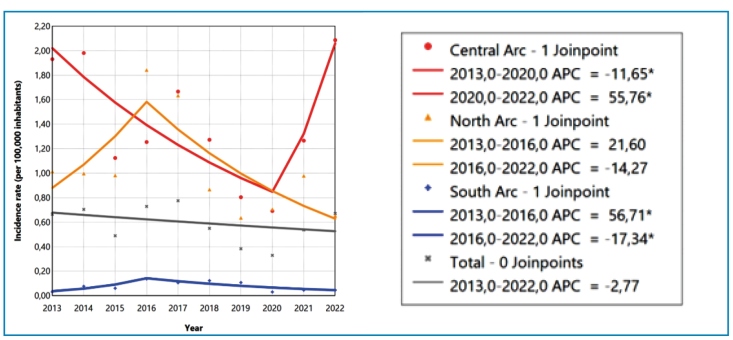
Temporal trend of visceral leishmaniasis incidence rate (per 100,000 inhabitants) in municipalities located in the border area of Brazil by Border Arc, 2013-2022. **Source:** prepared by the authors from Datasus data.

The temporal trend in the incidence rate of VL was stationary (APC: -2.77, 95% CI: -12.44 to 6.52) in the border area between 2013 and 2022. When stratified by Border Arcs, a heterogeneous temporal pattern was observed in the incidence rate of VL over the study period. In the Central Arc, a significant decreasing trend predominated from 2013 to 2020 (APC: -11.65, 95% CI: -27.28 to -5.91), followed by a significant increasing trend from 2020 to 2022 (APC: 55.76, 95% CI: 6.70 to 101.96). In the South Arc, was observed an initial significant increase from 2013 to 2016 (APC: 56.71, 95% CI: 9.63 to 329.93), followed by a significant decreasing trend from 2016 to 2022 (APC: -17.34, 95% CI: -49.46 to -7.74). In contrast, both segments analyzed in the North Arc showed stationary trends, despite fluctuations in the incidence rates (2013-2016: APC: 21.60, 95% CI: -18.86 to 204.11, 2016-2022: APC: -14.27, 95% CI: -68.67 to 14.67) (Figure 1 and Table 2).

**TABLE 2:** Temporal trend of visceral leishmaniasis incidence rate (per 100,000 inhabitants) in municipalities located in the border area of Brazil by Border Arc, 2013-2022

Region	Segment	Incidence rate		APC (95% CI)	p-value	Trend
		Initial	Final			
North Arc	2013-2016	1.01	1.84	21.60 (-18.86 to 204.11)	0.213	Stationary
	2016-2022	1.84	0.65	-14.27 (-68.67 to 14.67)	0.135	Stationary
Central Arc	2013-2020	1.93	0.69	-11.65 (-27.28 to -5.91)	<0.001	Decreasing
	2020-2022	0.69	2.09	55.76 (6.70 to 101.96)	0.006	Increasing
South Arc	2013-2016	0.03	0.14	56.71 (9.63 to 329.93)	0.006	Increasing
	2016-2022	0.14	0.05	-17.34 (-49.46 to -7.74)	0.004	Decreasing
**Total**	**2013-2022**	**0.67**	**0.67**	**-2.77 (-12.44 to 6.52)**	**0.449**	**Stationary**

Source: prepared by the authors from Datasus data.

**FIGURE 2:**
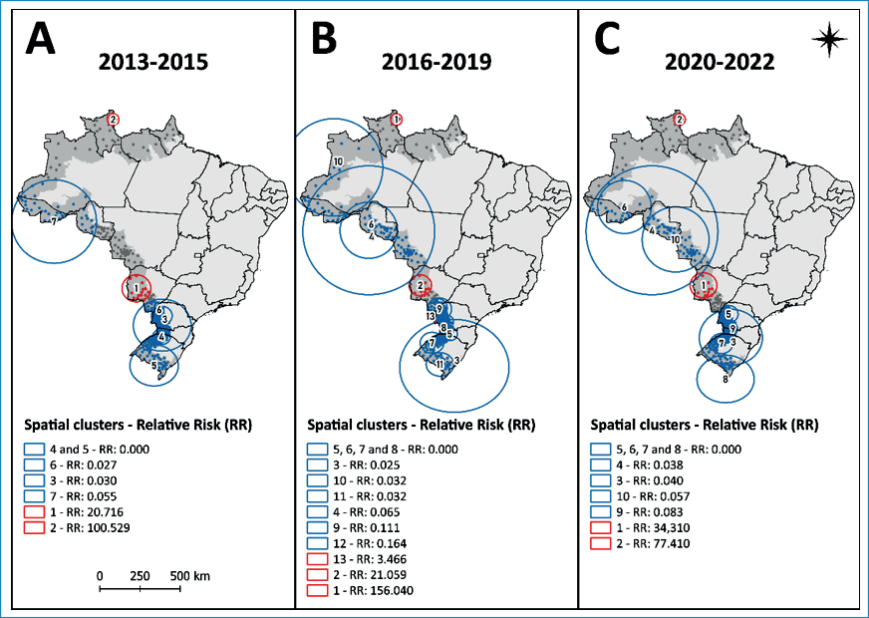
Spatial relative risk of visceral leishmaniasis in border-area municipalities of Brazil, stratified by periods: **(A)** 2013-2015; **(B)** 2016-2019; **(C)** 2020-2022**. Source:** prepared by the authors from Datasus data.

The spatial distribution of VL risk in Brazil’s border municipalities varied across the three defined time segments (Figure 2). In the first segment period (2013-2015), two high-risk clusters were identified: one in the North Arc (cluster 2, RR: 100.53, p < 0.001) and one in the Central Arc (cluster 1, RR: 20.71, p < 0.001). Five low-risk clusters (clusters 3 to 7) were observed in the South and North Arcs, with relative risks ranging from 0.00 to 0.05 (Figure 2A). During the second segment (2016-2019), three high-risk clusters were observed. In addition to the persistent clusters in the North Arc (cluster 1, RR: 156.04, p < 0.001) and Central Arc (cluster 2, RR: 21.06, p < 0.001), a new high-risk cluster (cluster 13) was detected in Foz do Iguaçu, a municipality located in the triple border region between Brazil, Paraguay, and Argentina, with a relative risk of 3.46 (p < 0.001). Ten low-risk clusters (clusters 3 to 13) were identified across all arcs, with RRs varying between 0.00 and 0.16 (Figure 2B). In the last segment (2020-2022), the same two high-risk areas remained: cluster 1 in the Central Arc (RR: 34.31, p < 0.001) and cluster 2 in the North Arc (RR: 77.41, p < 0.001). Eight low-risk clusters were identified in this period (clusters 3 to 10), with RRs between 0.00 and 0.08 (Figure 2C).

## DISCUSSION

In this study, higher VL incidence rates were identified in municipalities of the Central and North Arcs, with males and individuals aged 19 years or younger being the most affected groups. Although the overall temporal trend in the border area remained stationary, regional variations were observed, with decreasing trends in the North and South Arcs, and an increasing trend in the Central Arc. Spatial analysis revealed persistent high-risk clusters in the Central and North Arcs, as well as an emerging cluster in the South Arc.

The Brazilian border region faces a high burden of communicable diseases due to social vulnerability, limited healthcare access, and fragile surveillance systems, especially in remote municipalities^24^. Although the Unified Health System (*Sistema Único de Saúde*, SUS) has worked to implement control measures, barriers such as limited early diagnosis and low awareness among risk populations persist^25^. 

A general decline in VL incidence was observed in Brazil’s border region between 2013 and 2022. This decrease was most pronounced and statistically significant in municipalities of the Central Arc, with additional downward trends identified in the South and North Arcs after 2016. A possible explanation for the sharper decline in VL incidence from 2016 onwards is the strengthening of surveillance, diagnosis, and control structures. In Brazil, VL became a notifiable disease nationwide in 2016. Furthermore, in 2017 the regional Action Plan for the Surveillance and Control of Leishmaniases in the Americas (2017-2022) was approved, establishing goals to reduce VL incidence and lethality²⁶. These measures may have contributed to earlier detection and more effective interruption of transmission chains.

In border areas, intensified bi- or tri-national cooperation (Brazil and neighboring countries) and broader local intervention coverage^26^ may explain the differing decline patterns among the “arcs” (Central, South, and North). The faster decrease in the Central Arc could reflect more effective implementation of control measures compared to the South and North Arcs, where reductions occurred later, possibly due to greater spatial dispersion, lower surveillance capacity, and higher cross-border population mobility⁷.

Between 2020 and 2022, a significant resurgence in VL incidence was observed in the Central Arc, a region historically characterized by high transmission. This increase is possibly linked to disruptions caused by the COVID-19 pandemic, which impacted healthcare service delivery, particularly timely diagnosis, treatment, and case reporting^27^. These challenges are acknowledged in PAHO’s 2023-2030 Plan, which highlights persistent gaps in access to laboratory diagnosis, care, and rehabilitation for leishmaniasis, especially in the aftermath of the COVID-19 pandemic^5^ ^,^ ^27^. Additionally, border areas are marked by intense movement of people and goods, which can affect disease transmission and complicate surveillance efforts^7^. Reflecting this complexity, an increase in VL incidence was also observed in Paraguay between 2019 and 2022, whereas Brazil had maintained a sustained decline in incidence since 2015^5^. Notably, a concentration of cases has been identified along the border between Mato Grosso do Sul and Bolivia, underscoring the persistent transmission risk in border areas^28^.

A consistently persistent high-risk cluster was identified in the North Arc, state of Roraima, across all analyzed periods. In this context, studies have demonstrated a higher incidence of the disease in Brazil’s North region, where the rate was 1.36 cases per 100,000 inhabitants between 2001 and 2020^11^. In comparison, the present study found incidence rates ranging from 0.65 to 1.84 in the North Arc, with some municipalities forming a cluster with a relative risk of 156,04. Findings indicate that between 2007 and 2020 in the Legal Amazon, the cumulative VL incidence was 4.5 per 100,000 inhabitants, with high-risk clusters overlapping areas of deforestation^29^. Our results indicate a cumulative incidence rate of 9.42 (8.24 to 10.72) in municipalities within the North Arc, highlighting a concentration of VL cases along the Amazonian border region. In Colombia, VL has spread notably in socially vulnerable areas with limited healthcare access and poverty^16^.

In the South Arc, a high-risk cluster was identified between 2016 and 2019 in Foz do Iguaçu (Paraná), a tri-border area shared with Paraguay and Argentina. The persistent spread of VL in this region is supported by a combination of factors, including significant population movement, the presence of competent vectors, and animal reservoirs, particularly dogs^30^. Studies conducted in Foz do Iguaçu revealed a rising trend in canine VL seroprevalence, with rates ranging from 1.8% to 5.1% in 2012-2013 and reaching 46.25% in 2016^31^ ^,^ ^32^. Neighboring countries, Paraguay and Argentina, also face challenges with VL, largely influenced by environmental and social factors. Fragmented health systems and limited cross-border coordination underscore the importance of establishing integrated surveillance and control strategies in the region^15^ ^,^ ^24^ ^,^ ^33^.

Over the ten-year temporal analysis of VL incidence in the border region, there was no change in the location of low-risk areas, which remained predominantly concentrated from Acre to Mato Grosso (North and Central Arc) and from Paraná to Rio Grande do Sul (South Arc). Bordering countries with low-risk areas showed the same pattern, with cutaneous leishmaniasis presenting a decline from 2007 to 2022 in the Andean Region, and a stable trend in the Southern Cone between 2011 and 2022^5^. Argentina maintained low and stable VL cases between 2017 and 2022, with a slight increase following a low point in 2018, and approximately 10 cases reported in 2022. Despite the currently low risk, attention must be given to the ongoing spread of canine leishmaniasis caused by *L. infantum* into new areas of the Southern Cone^34^.

In 2023, men aged 20 to 50 years remained the most affected group in the Americas, consistent with the pattern observed in Brazil, which accounts for 91% of VL cases. However, our findings revealed a higher cumulative incidence among children and adolescents aged 19 years or younger. A similar epidemiological profile has been observed in Colombia and Paraguay, with a noticeable increase in cases among children under five years of age^35^. Findings indicate elevated incidence rates among children under six years of age, with 77.1% in rural areas of Pará state and 80.2% in the Northeast region^36^. Similarly, a study conducted in public hospitals in Portugal found that 60% of VL cases occurred in children under five^37^. Children and adolescents are particularly vulnerable to VL infection due to increased exposure to vector-prone environments and their still-developing immune systems^4^. Therefore, implementing grassroots health education initiatives targeting both children and the general public is essential to raise awareness of the disease and reduce risky behaviors^7^.

This study has some data-related limitations, including the use of secondary sources that may contain inconsistencies and missing information. Additionally, underreporting, particularly in remote areas with limited healthcare access, may lead to an underestimation of the true incidence rates^7^. Another consideration is that the COVID-19 pandemic may have caused disruptions in health services and surveillance between 2020 and 2022, potentially affecting case reporting and trend analysis^27^. Regional differences in incidence rates likely reflect disparities in surveillance capacity and healthcare access, especially in border municipalities^7^ ^,^ ^14^. A limitation of the temporal modeling is that it assumed a standard Poisson variance structure, which does not account for potential overdispersion and may affect the precision of the estimated trends.

Although this ecological study does not permit causal inferences at the individual level, it provides valuable insights into the spatial and temporal patterns of the disease over a 10-year period. These insights, based on an analysis adjusted to border areas, are crucial for guiding and optimizing public health interventions. Moreover, the observed regional differences in incidence rates emphasize the importance of considering disparities in surveillance capacity and healthcare access, especially in border municipalities. For future studies, it is recommended to use finer units of analysis, such as submunicipal levels (e.g., zip-code areas or individual spatial data), to more accurately detect spatial clusters that might be overlooked in municipal-level analyses, thus enabling more targeted and effective public policy interventions.

This study highlights the importance of continuous and integrated surveillance in border areas, supported by public health policies that address both local specificities and broader regional dynamics. Cross-border collaboration and information sharing are essential for effectively mitigating the risks associated with VL and for strengthening health system responses. The observed spatial patterns underscore the need for targeted interventions in high-risk areas, reinforcing that surveillance and control strategies must be tailored to the unique epidemiological and contextual conditions of each region. These findings can inform future research and support the development of more effective, context-sensitive public health policies aimed at reducing disease incidence and improving health outcomes in affected communities.

## Data Availability

The research data are available upon request.
